# Percutaneous Cryoablation of Elastofibroma Dorsi in a 73-Year-Old Woman: A Novel Minimally Invasive Alternative

**DOI:** 10.7759/cureus.99296

**Published:** 2025-12-15

**Authors:** David Mina, Vaiva Gustainyte, Shree Venkat, Wei Lue Tong, Hakob Kocharyan

**Affiliations:** 1 Interventional Radiology, Moffitt Cancer Center, Tampa, USA; 2 Diagnostic Imaging and Interventional Radiology, Moffitt Cancer Center, Tampa, USA

**Keywords:** cryoablation, elastofibroma dorsi, interventional radiology, minimally invasive, pneumodissection, soft-tissue tumor

## Abstract

Elastofibroma dorsi (EFD) is a benign soft-tissue pseudotumor typically managed with surgical excision. Minimally invasive ablation techniques have not been previously described for this entity. We report the case of a 73-year-old active woman who presented with a left infrascapular mass confirmed on biopsy as an elastofibroma. To preserve shoulder mobility and avoid extensive surgery, CT-guided percutaneous cryoablation was performed using three cryoprobes with pneumodissection to protect adjacent soft tissues. The procedure was well tolerated, and the patient was discharged the following day. At one-month follow-up, she reported significant improvement of shoulder discomfort, reduction of the palpable lump, and full return to activity. This represents, to our knowledge, the first reported instance of EFD successfully treated with percutaneous cryoablation. This minimally invasive approach appears to offer an effective, low-morbidity alternative to surgical excision for selected patients, preserving function and reducing recovery time.

## Introduction

Elastofibroma dorsi (EFD) is a benign, slow-growing soft-tissue lesion typically located deep to the scapula in elderly individuals, particularly women. It consists of elastic fibers, collagen, and adipose tissue, usually found between the inferior scapula and the chest wall. While many cases are asymptomatic, some patients experience discomfort or pain during shoulder motion. The lesion’s characteristic imaging appearance - a striated pattern of alternating fat and fibrous tissue - has been well described in radiologic literature, and its bilateral or multifocal presentation is not uncommon.

Surgical excision remains the standard treatment for symptomatic lesions, achieving definitive cure but associated with higher morbidity and longer recovery compared to minimally invasive approaches. Image-guided cryoablation has emerged as a minimally invasive option for managing benign and malignant musculoskeletal tumors with low morbidity and precise targeting under imaging guidance. To date, there have been no reports describing its application for EFD. We present what appears to be the first such case.

## Case presentation

A 73-year-old Caucasian woman presented with a recently discovered left subscapular mass associated with mild-to-moderate pain radiating to the left deltoid and biceps, which worsened with shoulder extension. She reported a left-sided infrascapular mass with no corresponding abnormality on the right, consistent with a unilateral presentation. Physical examination demonstrated a palpable, firm mass inferior to the scapula with discomfort on overhead extension and behind-the-back reach. She remained physically active, participating in Pilates several times weekly, but noted limitation with overhead extension and behind-the-back reach due to discomfort (pain 4/10 with motion). She had no significant comorbidities, was a lifelong non-smoker, and denied systemic symptoms.

**Figure 1:**
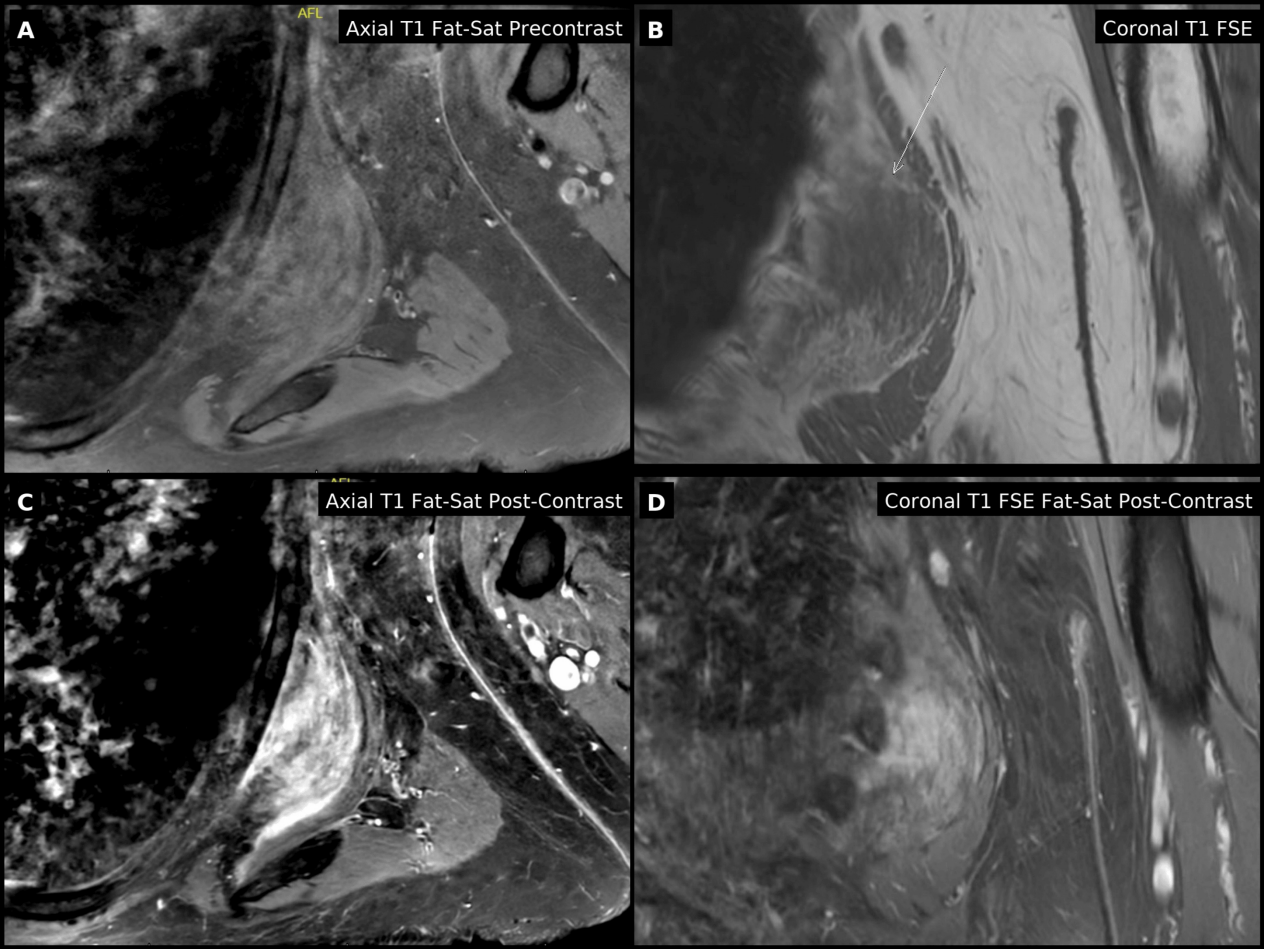
Pre-procedure MRI of elastofibroma dorsi (A) Axial T1 fat-saturated pre-contrast image showing a well-defined, striated soft-tissue mass deep to the left scapula, consistent with elastofibroma. (B) Coronal T1 FSE image demonstrating the lesion (arrow) between the latissimus dorsi and serratus anterior muscles. (C) Axial T1 fat-saturated post-contrast image showing mild peripheral enhancement. (D) Coronal T1 FSE fat-saturated post-contrast image confirming the characteristic alternating fibrous and fatty pattern.

MRI of the left scapula revealed a heterogeneous, striated soft-tissue mass measuring approximately 6.7 × 6.2 × 2.0 cm, located between the serratus anterior and latissimus dorsi muscles, consistent with EFD (Figure 1). The lesion demonstrated alternating fibrofatty signal intensity with mild peripheral enhancement. Scapular radiographs demonstrated a subtle lucent lesion with surrounding sclerosis but no acute bony abnormality. Core needle biopsy was performed to exclude atypical soft-tissue tumors, as imaging, while characteristic, cannot fully rule out malignancy. The biopsy sample provided adequate tissue for evaluation and demonstrated the characteristic elastic fibers, consistent with EFD.

After multidisciplinary discussion, percutaneous cryoablation and surgical resection were presented as potential treatment options. The patient opted for the less invasive percutaneous approach.

Procedural technique

**Figure 2:**
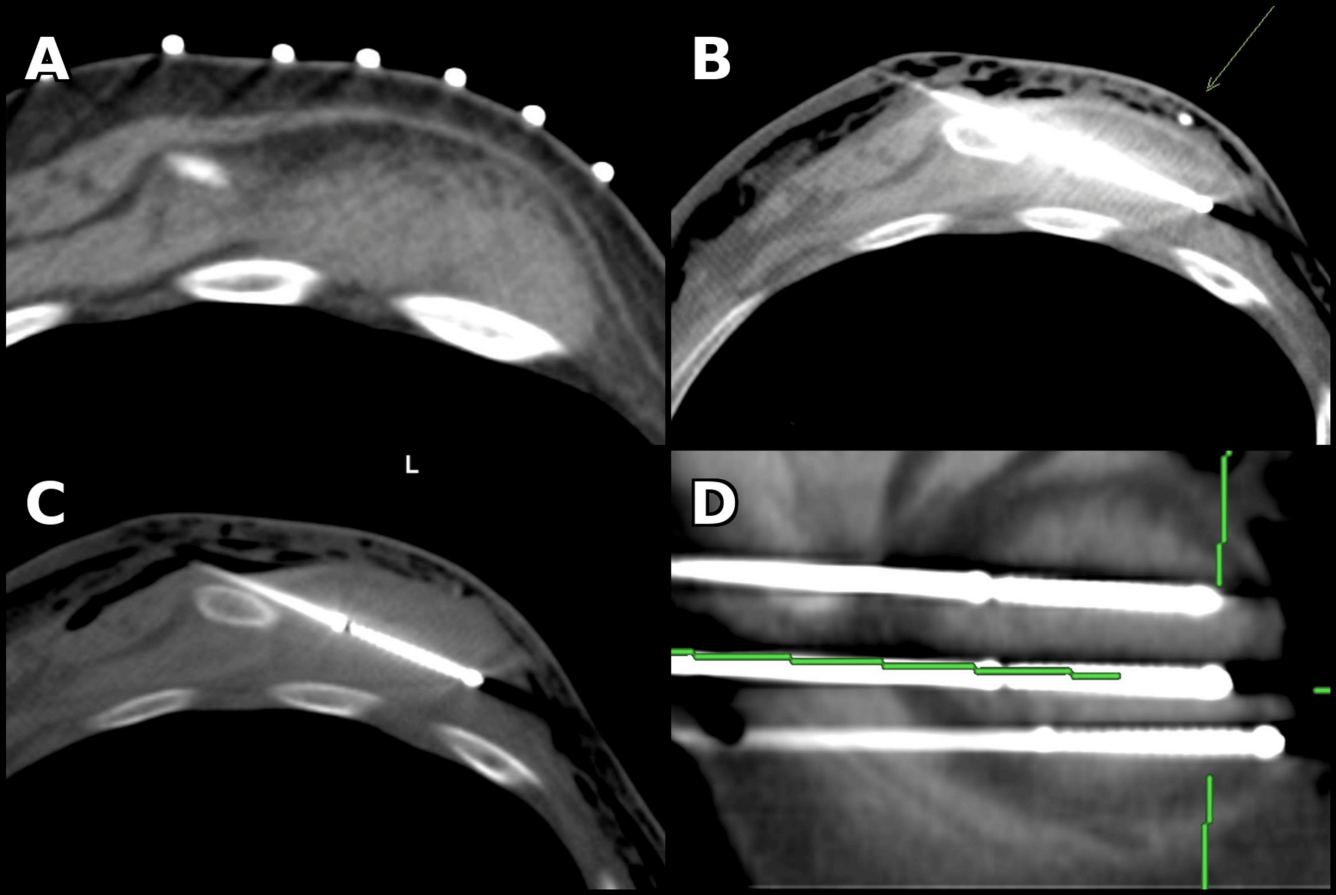
CT-guided percutaneous cryoablation of elastofibroma dorsi (A) Axial CT with grid marker used for probe trajectory planning. (B) Axial CT showing 5 Fr Yueh needle (arrow) used for pneumodissection to protect adjacent soft tissues. (C) Axial CT demonstrating probe placement within the lesion with early ice ball formation. (D) Sagittal reformatted CT image showing all three cryoprobes positioned across the mass.

Under general anesthesia and CT guidance, the patient was positioned in right lateral decubitus. Three Boston Scientific IceForce cryoprobes were placed within the lesion (Figure 2). Pneumodissection was performed using approximately 200 mL of air via a 5 Fr Yueh needle to displace and protect adjacent surrounding structures (Figure 2B). Hydrodissection was considered; however, pneumodissection was selected due to the lesion’s location and the operator’s preference for greater soft-tissue displacement in this region. Two freeze-thaw cycles were performed (10-minute freeze/eight-minute thaw) with intermittent CT imaging confirming that the ice ball encompassed the lesion with an adequate margin. Estimated blood loss was minimal, and the patient tolerated the procedure well without complication. She was observed overnight and discharged home the next day.

Outcome and follow-up

The post-procedural course was uneventful. The patient experienced mild transient soreness, which resolved within several days. At one-month follow-up, she reported significant resolution of shoulder discomfort, with pain decreasing from 4/10 pre-procedure to 2/10, along with a reduction of the palpable lump and full return to activity, including Pilates. No imaging was obtained at this visit, with follow-up imaging planned at six months. No delayed complications were observed. 

## Discussion

This case highlights the successful use of percutaneous cryoablation for symptomatic EFD, demonstrating that image-guided ablation can be a safe and effective alternative to surgery in select patients. EFD is a benign soft-tissue pseudotumor typically located deep to the inferior scapula, and its characteristic imaging appearance - a heterogeneous, striated pattern of fat and fibrous tissue - is well established in the literature [1-4]. The lesion is often bilateral and may be underrecognized on imaging studies, with reported prevalence rates as high as 2% on chest CT in elderly populations [4].

Surgical excision has traditionally been considered curative, but reported complication rates approach 17%, primarily due to seroma formation, hematoma, and delayed wound healing [5]. However, published reports describe considerable variability in seroma rates, reflecting differences in surgical technique and postoperative management. Recurrence after surgical excision is rare, whereas recurrence rates following cryoablation remain unknown due to the absence of long-term data. For patients seeking a less invasive option that preserves shoulder mobility and reduces postoperative morbidity, percutaneous cryoablation offers a compelling therapeutic choice.

Cryoablation provides several technical and clinical advantages, including precise control of ablation margins, real-time visualization of the ice ball, and relative preservation of surrounding tissue. Prior studies have confirmed its safety and efficacy in both benign and malignant soft-tissue tumors, with major complication rates typically below 5% [6-9]. The adjunctive use of pneumodissection in our case further enhanced safety by protecting adjacent thoracic structures, illustrating how such techniques can expand the boundaries of image-guided therapy in anatomically complex regions.

Previous reports have primarily described surgical management of EFD [5,10], with no prior documentation of percutaneous ablation. At one-month follow-up, our patient reported significant pain relief and a return to full physical activity, consistent with outcomes observed after ablation of other benign musculoskeletal lesions. Longer-term follow-up will be important to assess durability, recurrence risk, and to refine patient-selection criteria. Given the benign nature of EFD and reports of recurrence after incomplete excision, the long-term durability of cryoablation remains uncertain and cannot be inferred from this single short-term outcome. An additional limitation of this report is the absence of post-procedure imaging beyond intra-procedural CT monitoring, as no early follow-up imaging was obtained; this limits assessment of immediate radiologic response. Although no complications occurred in this case, potential risks of cryoablation in the infrascapular region include pneumothorax, neuropraxia, and injury to adjacent musculature. Ideal candidates for cryoablation are symptomatic patients seeking a less invasive option with preservation of shoulder function, whereas surgical excision remains preferable for very large lesions or when diagnostic certainty is limited.

To our knowledge, this represents the first published case of EFD successfully treated with percutaneous cryoablation. This technique may represent a promising addition to the minimally invasive treatment spectrum for benign chest-wall and subscapular tumors, providing rapid recovery and excellent functional outcomes while minimizing surgical morbidity. 

## Conclusions

Percutaneous cryoablation offers a promising, minimally invasive alternative to surgery for symptomatic EFD. It allows preservation of shoulder function, reduced morbidity, and rapid recovery.

The present case underscores the importance of multidisciplinary collaboration in managing complex benign and soft-tissue lesions. Early involvement of interventional radiology can provide minimally invasive alternatives to surgery, especially in patients with limited organ reserve or a preference for shorter recovery times. As image-guided therapies continue to evolve, their integration into comprehensive musculoskeletal care pathways will likely expand. Given the single-patient nature of this report and the short-term follow-up, no conclusions can be drawn regarding long-term efficacy or recurrence. Larger, multi-institutional experiences will be needed to define optimal patient selection and assess the durability of cryoablation for EFD. Further reports and long-term follow-up will be essential to establish procedural standards and refine patient selection criteria.
